# A two-arm parallel double-blind randomised controlled pilot trial of the efficacy of Omega-3 polyunsaturated fatty acids for the treatment of women with endometriosis-associated pain (PurFECT1)

**DOI:** 10.1371/journal.pone.0227695

**Published:** 2020-01-17

**Authors:** Ibtisam M. Abokhrais, Fiona C. Denison, Lucy H. R. Whitaker, Philippa T. K. Saunders, Ann Doust, Linda J. Williams, Andrew W. Horne

**Affiliations:** 1 MRC Centre for Reproductive Health, University of Edinburgh, Edinburgh, United Kingdom; 2 Centre for Inflammation Research, University of Edinburgh, Edinburgh, United Kingdom; 3 Usher Institute, University of Edinburgh, Edinburgh, United Kingdom; Poissy-Saint Germain Hospital/Versailles Saint Quentin University, FRANCE

## Abstract

**Background:**

Endometriosis is defined by the presence of endometrial-like tissue (lesions) outside the uterus, commonly on the pelvic peritoneum. It affects 6–10% of women and is associated with debilitating pelvic pain. Current management options are often unsatisfactory. Omega-3 polyunsaturated fatty acids (O-PUFA) have the potential to reduce the painful symptoms associated with endometriosis, reduce lesion size, preserve the patient’s ability to conceive, and have minimal side effects. We performed a two-arm, parallel double-blinded randomised controlled trial to inform the planning of a future multicentre randomised controlled trial to evaluate the efficacy of O-PUFA for endometriosis-associated pain.

**Objectives:**

The primary objectives of the trial were to assess recruitment and retention rates. The secondary objectives were to determine the acceptability to women of the proposed methods of recruitment, randomisation, treatments and questionnaires, to estimate the variability in the proposed primary endpoints to inform the sample size calculation and to refine the research methodology for the future definitive trial.

**Methods:**

We recruited women with endometriosis from June 2016 to June 2017 and randomised them to eight weeks of treatment with O-PUFA or olive oil. Pain scores and quality of life questionnaires were collected at baseline and eight weeks. We calculated the proportion of eligible women randomised, and of randomised participants who were followed up to eight weeks. Acceptability questionnaires were used to evaluate women’s experiences of the trial.

**Results:**

The proportion of eligible participants who were randomised was 45.2% (33/73) and 81.8% (27/33) completed the study. The majority of participants described their overall trial experience favourably and there were no adverse events in either group.

**Conclusion:**

Our pilot trial supports the feasibility of a future larger trial to definitively evaluate the efficacy of O-PUFA for endometriosis-associated pain.

**Trial registration:**

The trial was registered on the ISRCTN registry (registration number ISRCTN44202346).

## Introduction

Endometriosis is a chronic oestrogen-dependent inflammatory condition that affects around 176 million women worldwide [1, 2]. It is defined by the presence of endometrial-like tissue (‘lesions’) outside the uterus, and is associated with debilitating pelvic pain and infertility. It is a complex, heterogeneous disorder, poorly understood, and the aetiology remains unknown. Management options for endometriosis-associated pain include surgical removal of lesions and medical treatment with ovarian suppressive drugs [3, 4]. However, the recurrence of endometriosis-associated pain is up to 50% within five years following surgery [5], and drug treatments are contraceptive and/or have menopausal-like side-effects and may not fully resolve pain [6].

Oral Omega-3 polyunsaturated fatty acids (O-PUFA; eicosapentaenoic acid [EPA] and docosahexaenoic acid [DHA]) [7] have potential to (i) reduce the painful symptoms associated with the condition, (ii) reduce lesion size, (iii) preserve the patients’ ability to conceive while on medication, and (iv) have no, or limited, side effects. The Food and Drug Association (FDA) has approved a “qualified health claim” for O-PUFA for lowering plasma triglycerides and approved several high dose formulations as drugs [8]. However, whilst there are mechanistic data to support a lipid-mediated mechanism for endometriosis [9], there is only anecdotal evidence of benefit from O-PUFA and no evidence from randomised controlled trials in this population.

O-PUFAs play a role in the regulation of prostaglandins and cytokines, factors critical to endometriosis, by competing with Omega-6 PUFA to produce anti-inflammatory lipid mediators [6]. Intake of food with a high content of O-PUFA has been shown to have an anti-inflammatory effect in conditions such as atherosclerosis [10]. Importantly, it has been shown that O-PUFAs can serve as substrates for formation of a wide range of mediators implicated in resolution of inflammation [11–15].

In a rat model of endometriosis, O-PUFA reduced lesion size and local prostaglandin and cytokine production [15]. Administration of D-series resolvins (DHA metabolites) also reduced vaginal hyperalgesia [14]. In an endometriosis model using Fat-1 mice, in which Omega-6 can be converted to O-PUFA, the number of induced lesions were significantly less in Fat-1 mice compared to controls [13] and inflammatory mediators such as IL-6 and Cox-2 levels were reduced [12]. In in-vitro studies, using endometrium from women with endometriosis, it has been shown that O-PUFA has a suppressive effect on endometrial cell survival [11], corroborating the animal data.

A large, prospective cohort study investigated dietary exposure and endometriosis risk in women, and concluded that increasing O-PUFA intake might reduce the risk of endometriosis [16]. A dietary modification and supplementation trial, which included O-PUFA, reduced pain scores in women with endometriosis [17]. A low ratio of O-PUFA to Omega-6 PUFA intake is also reported to correlate with painful menstruation in women with endometriosis [18], with fish oil supplementation significantly reducing pain scores in adolescents with menstrual pain [19].

We believe that it is important to determine the true value of O-PUFA for the control of, or potential elimination of, endometriosis-associated pain. A definitive evaluation of the efficacy of O-PUFA in the management of endometriosis-associated pain will require a large, multicentre randomised controlled trial (RCT). We therefore performed a two-arm, parallel double-blind randomised controlled pilot trial to inform the planning of a future multicentre RCT to evaluate the efficacy of O-PUFA for endometriosis-associated pain.

## Materials and methods

### Study design

**Fig 1:**
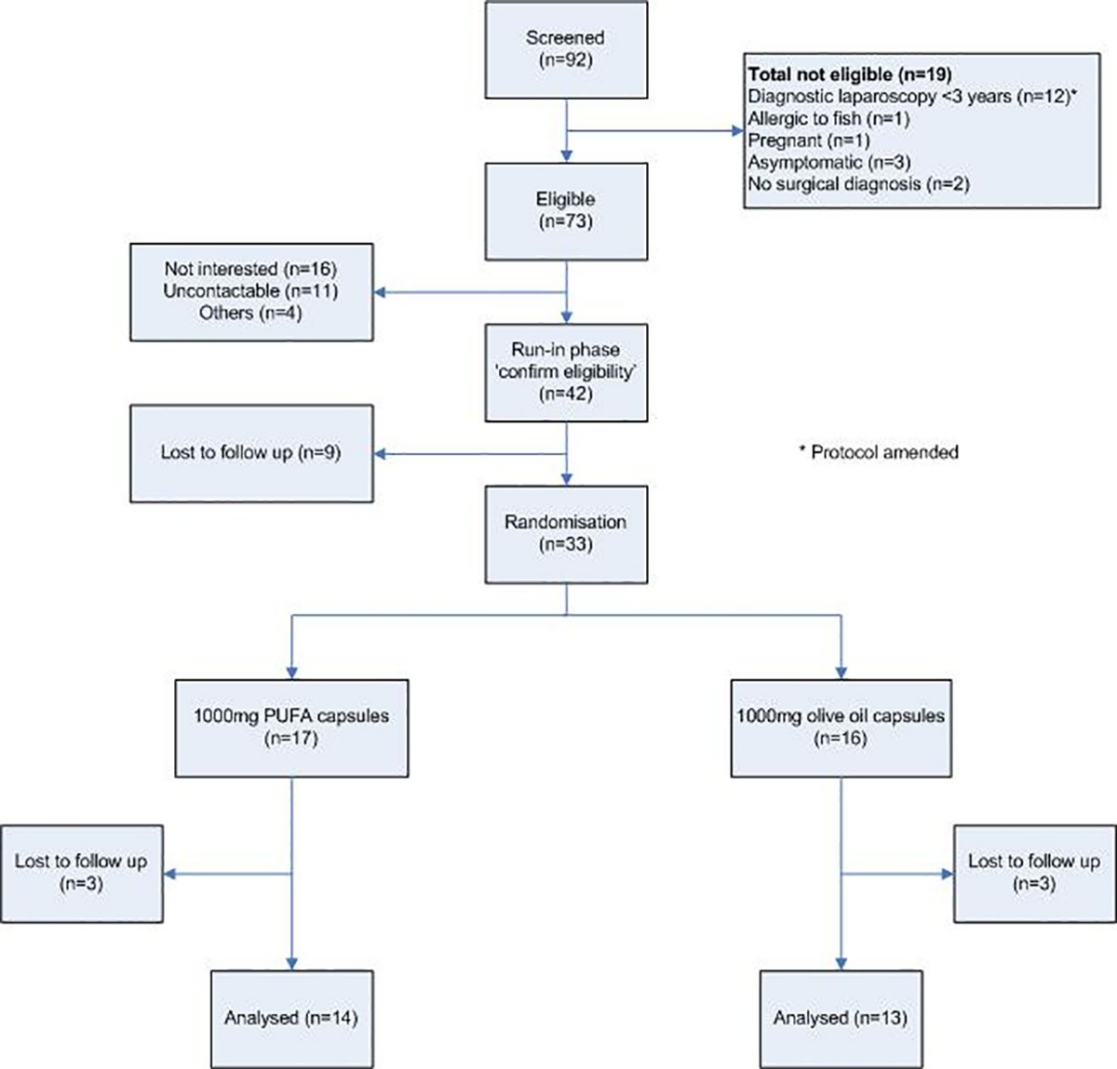
Trial consort diagram.

We performed a two-arm, parallel, double-blind randomised controlled pilot trial. Women with a surgical diagnosis of endometriosis and associated pelvic pain were recruited from gynaecology outpatient clinics, gynaecology wards and day surgery units within the Royal Infirmary of Edinburgh over a 12-month period from 30^th^ June 2016 to 16^th^ March 2017. Data Collection was completed on 7^th^ June 2017 (Fig 1). The trial protocol has been published online [20]. Ethical approval was obtained from the Scotland A Research Ethics Committee (LREC 16/SS/0010) on 02/02/2016 and informed written consent was obtained from all participants.

### Inclusion criteria

Women were eligible for inclusion if they were as follows:

- Aged between 18 and 50 years.
- Pelvic pain of > 3 months
- Pain located within the true pelvis (between or below the iliac crests)[21]
- Endometriosis diagnosed macroscopically by laparoscopy (performed at least two weeks prior to enrolment)
- A numerical rating average ‘worst’ pain score (NRS) of greater than or equal (≥) to four (see below for details).

### Exclusion criteria

Women were excluded if they had one or more of the following:

- Unable to take/allergic to fish/O-PUFA/peanuts/soyabean
- Insulin-dependent diabetes
- Pregnancy
- Taking anticoagulants
- Breast feeding
- Unable to give informed consent

### Sample size

The aim of this pilot was to establish feasibility of a future trial—it was therefore designed primarily to explore recruitment and retention rates. To achieve this, the aim was to recruit as many women as possible over a 12-month period. Based on estimates of the number of eligible patients, we postulated that we would recruit four to five patients per month (and recruit 40 patients in 12 months). Based on a recruitment rate of 50%, we estimated that 40 participants would give a 95% confidence interval (CI) of 39–61% (using the Normal approximation since the denominator is ≥50).

### Screening

Eligibility for randomisation was based on the worst of four weekly numerical rating scale (0–10; NRS) pain scores collected by text messaging (‘run-in phase’). A score of four out of ten in two or more weeks was required. Texts were sent to the woman’s mobile phone, asking about average and worst pain, respectively, and the woman was asked to reply to the text message with her pain score, rating it from 0 for no pain at all, to 10 being worst pain imaginable. To capture known cyclicity in pain, the texts were sent once per week during the eligibility phase (weeks ‘minus four’ to ‘minus one’). If a woman had an NRS of greater than or equal to ≥ four in two or more of the pre-randomisation run-in weeks of the study, she was considered to be eligible for randomisation.

### Randomisation

The randomisation scheme was generated using tables, drawn up by an independent statistician, with 1:1 allocation ratio using blocks of four and six in a random pattern. The allocated treatment codes were sealed in consecutively numbered opaque envelopes. When a woman was randomised into the study, she received the treatment indicated in the next available envelope.

### Interventions

Participants were randomly assigned to the intervention (Omega-3-acid ethyl ester PUFA filled capsules, one capsule (1000mg) twice a day) or a visually identical comparator (olive oil, soft gelatin capsules, one capsule twice a day) for eight weeks. The allocated treatments were taken for eight weeks. O-PUFA capsules have been used clinically in previous trials (post-myocardial infarction and for hypertriglyceridemia) at doses of 1000-4000mg daily (in divided doses of 1000mg or 2000mg) [7]. However, high dosage (i.e. 4000mg) has been associated with a moderate increase in post-operative bleeding so we chose to pilot a lower dose and frequency of one capsule (1000mg) twice a day. We approached the Medicines and Healthcare products Regulatory Agency (MHRA) for advice regarding the classification of O-PUFA and olive oil. As both O-PUFA and olive oil capsules are ‘food supplements’, our trial was not deemed a Clinical Trial of an Investigational Medicinal Product (CTIMP).

### Co-primary objectives

Recruitment and retention rates.

### Secondary objectives

- Estimation of the variability of responses to inform the primary outcome and sample size of the future RCT.
- Acceptability of proposed methods of recruitment, randomisation, treatments and questionnaires.

### Questionnaires

Participants were given the questionnaires below (see published protocol for further details [20]) to complete at baseline and eight weeks. Baseline demographic and clinical characteristics of the participants were also recorded at baseline.

- Brief Pain Inventory (BPI)
- 12-Item Short-Form Health Survey (SF-12)
- Pain Catastrophizing Questionnaire (PCQ)
- Pain DETECT^TM^
- Sexual Activity Questionnaire (SAQ)
- Brief Fatigue Inventory (BFI)
- General Health Questionnaire-12 (GHQ-12)
- Work and Productivity Impairment
- Questionnaire-specific Health Problem Version 2.0 (WPAI-SHP)

An additional acceptability questionnaire was completed at the end of the study only. This questionnaire included questions about whether participants believed that they were receiving O-PUFA or olive oil, the acceptability of the allocated medication/ treatment regimens (and compliance) and on the acceptability of completing trial-specific procedures including the questionnaires. All questionnaires were anonymised and self-completed in private.

### Treatment diaries

Participants were provided with a treatment diary at the same time as their medication pack was dispensed. All medications other than the trial treatment taken during the treatment phase of the study were recorded in a treatment diary. This included prescription and non-prescription treatments, such as oral analgesics, contraceptives, vitamins, topical preparations and herbal preparations.

### Adverse events

Participants collected information about adverse events in their treatment diaries. They were also instructed to contact the clinical trial team at any time after consenting to join the trial if they had an event that requires hospitalisation or an event that resulted in persistent or significant disability or incapacity.

### Participant log

We kept an anonymised electronic log of women who fulfilled the eligibility criteria, women who were invited to participate in the study, women who were recruited and women who left the trial early. Reasons for non-recruitment (e.g. non-eligibility, refusal to participate, administrative error) were also recorded. We attempted to collect reasons for non-participation from women who declined to take part after previously providing contact details. During the course of the study, we attempted to document reasons for withdrawal from the study and loss to follow-up.

### Statistical analysis

A statistical analysis plan was agreed with the trial statistician (Dr Linda Williams, University of Edinburgh) prior to a database lock. Estimates of recruitment and retention were to be calculated with their 95% Confidence Interval (CI). Where the denominator is small (<50), exact binomial confidence intervals will be calculated. Where the denominator is large the Normal approximation will be used. Change from baseline for each of the questionnaires and sub-questions were calculated for each patient, and these changes were compared between treatments using the t-test. Where the change from baseline was not normally distributed, the change was transformed on the log scale, and this was compared between treatments. All tests were by intention-to-treat and two-sided, with a 1% level of significance to make some allowance for multiple testing. Only patients with baseline and follow-up at eight weeks were included. Due to the small sample size, no attempts were made to impute missing data. Statistical analysis was performed using Microsoft Excel (ME) and SPSS software version 23. The number and nature of unanswered questions was recorded and explored.

### Trial sponsors

The trial was co-sponsored by the University of Edinburgh and NHS Lothian.

## Results

### Baseline characteristics

Table 1 presents the participants’ characteristics per group. The majority of participants had a menstrual cycle, more than three-quarters of them had attained a college/university degree and only one participant was non-Caucasian (Asian). More than half of the participants (57%) never smoked. Two thirds of them had never been pregnant. 79% of the recruited women had a diagnosis of superficial peritoneal endometriosis. Less than half of the participants required contraception and the levonorgestrel- releasing intrauterine system was the most popular method.

**Table 1:** Baseline characteristics.

		O-PUFA		Olive Oil Capsules	
		N	%	N	%
		17	51.5	16	48.5
**Age**	Mean	35.43		36.08	
	Std. Deviation	8.57		9.59	
	Range	21–48		24–49	
**BMI (kg/m**^**2**^**)**	Mean	27.86		23.95	-
	Std. Deviation	7.89		4.29	
	Range	18.3–41.1		16.9–33.4	
**Highest education received**	Primary school	0	0	0	0
	Secondary school	3	11.1	3	11.1
	College/university	11	40.7	10	37
**Ethnicity**	Caucasian	14	51.9	12	44.4
	Other (Asian)	0	0	1	4
**Smoking**	Smoker	3	10.7	1	3.6
	Ex-smoker	4	14.3	4	14.3
	Never smoked	8	28.6	8	28.6
**Diet habit**	Vegetarian	0	0	0	0
	Seafood	0	0	0	0
	Vegetarian and seafood	2	14.3	2	15.4
	Others	12	85.7	11	84.6
**Parity**	None	10	37	8	29.6
	1 or more	4	14.8	5	18.5
**Periods**	Yes	11	40.7	4	14.8
	No	3	11.1	9	33.3
**Pain during periods**	Yes	11	40.7	7	25.9
	No	3	11.1	6	22.2
**Pain during intercourse**	Yes	11	44	9	36
	No	3	11.1	4	14.8
**Previous gynaecological operations (other than first laparoscopy)**	>1	4	28.5	7	53.8
	>2	3	21.4	3	23
	>3 or more	7	50	3	23
**Type of endometriosis**	Superficial peritoneal	10	41.7	9	37.5
	Ovarian	1	4.2	1	4.2
	Deep	2	8.3	1	4.2
**Contraception**	Patch	0	0	0	0
	Nexplanon	0	0	0	0
	COCP	1	3.7	1	3.7
	Depo-Provera	0	0	1	3.7
	POP	0	0	2	7.4
	Contraceptive ring	0	0	0	0
	LNG-IUS	4	14.8	4	14.8
	Other	1	3.7	1	3.7
	Any used	5	18.5	7	25.9

COCP: combined oral contraceptive pill, POP: progesterone only contraceptive pill, LNG-IUS: levonorgestrel- releasing intrauterine system. Deep endometriosis is defined as >5mm infiltration [22]

### Recruitment and retention rates

The proportion of eligible participants who were randomised over 12 months was 45.2% (33/73, 95% CI 33.5, 57.3%)) and 81.8% (27/33, 95% CI (64.5, 93.0%)) completed the study. Seventeen participants were randomised to receive O-PUFA group and 16 participants were randomised to receive olive oil capsules. Examples of reasons for non-participation included ‘trying to conceive’, ‘strict vegetarian and will not accept fish oil, want only olive oil’, and ‘already taken the fish oil without much help’. Six participants were lost to follow-up, three from each treatment group.

### Estimation of variability of responses to inform the primary outcome of the future definitive trial

Supplementary tables (S1–S8 Tables) summarise the variability in responses to the questionnaires. There was no statistically significant difference in pain scores or quality of life outcomes in the two treatment groups but there was a trend toward improvement in pelvic pain and quality of life scores in both treatment arms over the eight week intervention points. For the secondary outcome measures, estimates of treatment effects were presented with 95% two-sided CI. P-values were reported from two-sided tests at the 5% significance level, although as secondary endpoints these were mostly under-powered. A two-sided P-value of ≤ 0.01 was considered statistically significant.

**Fig 2:**
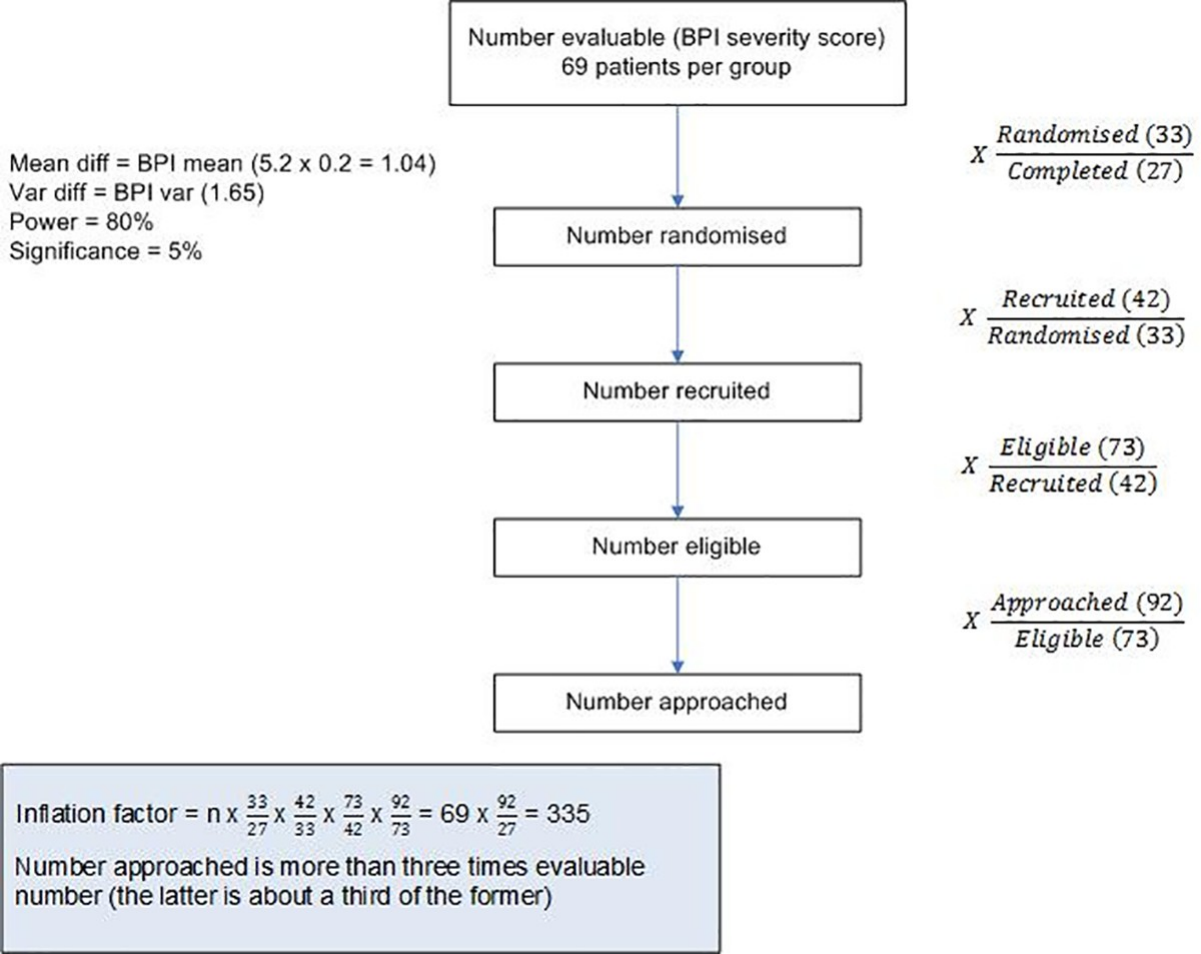
Example of sample size calculation for future definitive trial based on the BPI severity and interference scores.

In order to calculate the required sample size for a future RCT, we assumed that our primary outcome measure was the BPI severity score. From our results, we estimated a pooled standard deviation (SD) of 2.08. In order to detect a difference of 1 unit on the BPI scale, which is approximately a fall of 20% from the baseline mean, with a power of 80% and a significance level of 5%, we utilised the sample size equation for a difference in continuous data. This requires at least 69 analysable patients per group. Similarly, if we wished to use the interference score as the primary or co-primary outcome, the pooled SD is 2.18 and we would require 76 patients per group. However, we would also need to take loss to follow up into account (retention), and the recruitment rate in order to estimate the number of patients we would need to approach in order to achieve sufficient patients to achieve our primary outcome. A point estimate and 95% CI have been reported for recruitment and retention. As these figures (69 or 76) refer to the number of evaluable patients required, adjustments for retention will be made (Fig 2).

### Acceptability of interventions

The acceptability of the interventions was evaluated by calculating the proportion of participants who attended all visits and completed their treatments: 82.4% (95% CI 56.6, 96.2%) of participants (14/17) who received O-PUFA and 81.3% (95% CI 54.4, 96.0%) of participants (13/16) who received olive oil capsules attended all visits. The majority of participants described their overall trial experience favourably and rated it one or two on a five-point scale from positive to negative. Positive responses were recorded for the recruitment approach used, paperwork burden for participants and the experience of randomisation process of participants. Negative feedback was largely related to the size of the pills as some women experienced discomfort when swallowing the large capsules, time taken to complete the treatment diary and complexity of some of the questionnaires. The trial was not powered to detect difference in outcomes recorded by questionnaires.

### Adverse events

There was no report of any adverse event from any of the participant in either group.

## Discussion

Our pilot trial demonstrates that a future definitive RCT to determine the efficacy of O-PUFA for the treatment of women with endometriosis-associated pain is feasible. Feasibility is supported by the fact that, over a 12 month recruitment period, we were able to randomise 33 women (45.2% of those eligible) to O-PUFA or comparator and obtain completed eight week follow up data in 27 women (81.8%).

A recent comprehensive review of the recruitment and retention data of a cohort of 151 RCTs funded and published by the UK NIHR HTA Programme from 2004 to 2016 found that the average retention rate was 89% and the average proportion of eligible patients who are randomised was 70% [23]. Whilst the retention rates in our pilot were less than the published average retention rate of 89% (81.8%), we believe that this simply reflects the challenges of working with a target population who have a chronic pain condition. We will therefore inflate the sample size in our future RCT for to account for this level of attrition. The randomisation rates were also less than the published average of 70% (45.2%) and marginally lower than we had predicted (50%). These data are important and will allow us to estimate recruitment and randomisation more accurately in our proposed future trial, and determine the number of centres and the recruitment period that we will require. We are conscious that our trial had no patient and public involvement (PPI) and aware of data on PPI in clinical trials that indicates that PPI is likely to improve randomisation and retention of participants, especially if the PPI group includes people with lived experience of the health condition under study [24]. We will therefore ensure that we involve women with endometriosis and representatives from relevant patient organisations in all aspects of our future trial, including the early stages of the trial proposal and design.

We were pleased to note that analysis of the data regarding the delivery of the trial was generally favourable. The limited negative feedback that we received was largely related to trial medication (large size of capsules) and treatment diary completion. Whilst the delivery of the medication cannot be altered, the other feedback has alerted us to the need to develop the option of a more accessible and user-friendly online treatment diary.

The observed trend toward improvement in pelvic pain and quality of life scores in both treatment arms over the eight week intervention points to a strong placebo effect, which would require a much larger sample size, or the use of much smaller (and likely clinically irrelevant) effect sizes, to detect statistically significant differences between treatment groups. This suggests an effect from enhanced clinical interaction and trial participation on pain, psychological and physical well-being, and highlights a need for qualitative research to explore whether women experience a placebo effect which influences their experiences and reporting of pain and hence, potentially, trial outcomes. We are also aware that we used a potentially ‘active’ placebo. Olive oil contains oleocanthal which has been shown to act as a natural anti-inflammatory compound that has a potency and profile strikingly similar to that of ibuprofen [25]. We plan to use a more ‘inert’ substance as our placebo, that is unable to elicit an effect, our future trial.

## Conclusions

In conclusion, we are now ready to capitalise on our pilot trial and secure funding to perform a definitive large multicentre double-blind randomised controlled trial to determine the efficacy of O-PUFA for the treatment of women with endometriosis-associated pain.

## Supporting information

S1 Consort checklist CONSORT checklist for RCT protocols. (DOC)

S1 Table Results from secondary outcome measures–SF12. SF-12 version 2 scores range from 0–100, where low scores are bad and high scores are good.
(DOCX)

S2 Table Results from secondary outcome measures–BPI. BPI scores range from 0–10, where low scores are good and high scores are bad.
(DOCX)

S3 Table Results from secondary outcome measures–BFI. BFI scores range from 0–10, where low scores are good and high scores are bad.
(DOCX)

S4 Table Results from secondary outcome measures–GHQ. GHQ scores range from 0–12, where higher scores represent higher levels of mental distress.
(DOCX)

S5 Table Results from secondary outcome measures–WPAIQ. WPAIQ scores range from 0–100, where low scores are good and high scores are bad indicating greater impairment and less productivity.
(DOCX)

S6 Table Results from secondary outcome measures–PCQ. ^1^ PCQ rumination scores range from 0–16, where low scores are good and high scores are bad. ^2^ PCQ magnification scores range from 0–12, where low scores are good and high scores are bad. ^3^ PCQ helplessness scores range from 0–24, where low scores are good and high scores are bad. ^4^ PCQ total scores range from 0–52, where low scores are good and high scores are bad.
(DOCX)

S7 Table Results from secondary outcome measures–SAQ. ^1^ SAQ pleasure scores range from 0–18, where low scores are bad and high scores are good. ^2^ SAQ discomfort scores range from 0–6, where low scores are bad and high scores are good. ^3^ SAQ habit scores range from 0–3, where low scores are bad and high scores are good. ^4^ SAQ tired scores range from 0–3, where low scores are bad and high scores are good.
(DOCX)

S8 Table Results from secondary outcome measures–PDQ. PDQ scores range from 0–35, where low scores are good and high scores are bad.
(DOCX)

S1 Protocol (PDF)
