# Correction: A two-arm parallel double-blind randomised controlled pilot trial of the efficacy of Omega-3 polyunsaturated fatty acids for the treatment of women with endometriosis-associated pain (PurFECT1)

**DOI:** 10.1371/journal.pone.0230055

**Published:** 2020-02-27

**Authors:** Ibtisam M. Abokhrais, Fiona C. Denison, Lucy H. R. Whitaker, Philippa T. K. Saunders, Ann Doust, Linda J. Williams, Andrew W. Horne

There are errors in the Author Contributions for Ibtisam M. Abokhrais. The correct contributions for Ibtisam M. Abokhrais are: Formal analysis, Investigation, Project administration, Visualization, Writing–original draft.
