# Supplementary material for: A two-arm parallel double-blind randomised controlled pilot trial of the efficacy of Omega-3 polyunsaturated fatty acids for the treatment of women with endometriosis-associated pain (PurFECT1)
Source: PLoS One. 2020 Jan 17;15(1):e0227695. doi: 10.1371/journal.pone.0227695 (PMC6968860; doi:10.1371/journal.pone.0227695)
Supplement: S1 Table — SF-12 version 2 scores range from 0–100, where low scores are bad and high scores are good. (DOCX) [file pone.0227695.s002.docx]

**S1 Table. Results from secondary outcome measures – SF12**

|  | **Randomised Treatment** | | | | | | |  | | |
| --- | --- | --- | --- | --- | --- | --- | --- | --- | --- | --- |
|  | **PUFA** | | |  | **Olive Oil** | | |  |  |  |
|  | **N** | **Mean** | **SD** |  | **N** | **Mean** | **SD** | **Mean diff^1^**  **in change** | **95% CI** | **P-value** |
|  |  |  |  |  |  |  |  |  |  | **(t-test)** |
| **SF-12 (higher score = better)** | | | | | | | | | | |
| Physical_component_summary_baseline_ score | 14 | 2.58 | 0.59 |  | 13 | 2.65 | 0.76 | - | - | - |
| Physical_component_summary_8_weeks_score | 14 | 2.62 | 0.43 |  | 13 | 2.6 | 0.76 | - | - | - |
| Change from baseline (8 weeks-baseline) | 14 | 0.04 | 0.52 |  | 13 | -0.05 | 0.53 | 0.09 | (-0.33  **̶** 0.50) | 0.670 |
| Mental_component_summary_baseline_ score | 14 | 2.94 | 0.63 |  | 13 | 2.79 | 0.67 | - | - | - |
| Mental_component_summary_8_weeks_ score | 14 | 2.89 | 0.76 |  | 13 | 2.94 | 0.81 | - | - | - |
| Change from baseline (8 weeks-baseline) | 14 | -0.05 | 0.54 |  | 13 | 0.15 | 0.60 | -0.21 | (-0.66 ̶ 0.25) | 0.356 |

SF-12 version 2 scores range from 0-100, where low scores are bad and high scores are good.
