# Supplementary material for: A two-arm parallel double-blind randomised controlled pilot trial of the efficacy of Omega-3 polyunsaturated fatty acids for the treatment of women with endometriosis-associated pain (PurFECT1)
Source: PLoS One. 2020 Jan 17;15(1):e0227695. doi: 10.1371/journal.pone.0227695 (PMC6968860; doi:10.1371/journal.pone.0227695)
Supplement: S2 Table — BPI scores range from 0–10, where low scores are good and high scores are bad. (DOCX) [file pone.0227695.s003.docx]

**S2 Table. Results from secondary outcome measures – BPI**

|  | **Randomised treatment** | | | | | | |  | | |
| --- | --- | --- | --- | --- | --- | --- | --- | --- | --- | --- |
|  | **PUFA** | | |  | **Olive Oil** | | |  |  |  |
|  | **N** | **Mean** | **SD** |  | **N** | **Mean** | **SD** | **Mean diff in change** | **95% CI** | **P-value** |
|  |  |  |  |  |  |  |  |  |  | **(t-test)** |
| **BPI (higher score = worse)** | | | | | | | | | | |
| Pain severity baseline score | 14 | 4.92 | 2 |  | 13 | 5.21 | 2.50 | - | - | - |
| Pain severity week 8 score | 14 | 4.96 | 1.73 |  | 13 | 4.54 | 2.44 | - | - | - |
| Change from baseline (8 weeks-baseline) | 14 | 0.04 | 1.78 |  | 13 | -0.67 | 2.35 | 0.71 | (-0.93 - 2.35 ) | 0.383 |
| Pain interference baseline score | 14 | 4.57 | 2.33 |  | 13 | 5.83 | 2.55 | - | - | - |
| Pain interference week 8 score | 14 | 4.54 | 2.41 |  | 13 | 4.60 | 3.36 | - | - | - |
| Change from baseline (8 weeks-baseline) | 14 | -0.03 | 1.95 |  | 13 | -1.23 | 2.39 | 1.20 | (-0.52 **-** 2.92) | 0.164 |
| Change from baseline (8 weeks-baseline) (pain interference_diff_transformed) | 14 | 0.13 | 0.33 |  | 13 | -0.03 | 0.48 | 0.16 | (-0.17 - 0.49) | 0.322 |

BPI scores range from 0-10, where low scores are good and high scores are bad.
