# Supplementary material for: A two-arm parallel double-blind randomised controlled pilot trial of the efficacy of Omega-3 polyunsaturated fatty acids for the treatment of women with endometriosis-associated pain (PurFECT1)
Source: PLoS One. 2020 Jan 17;15(1):e0227695. doi: 10.1371/journal.pone.0227695 (PMC6968860; doi:10.1371/journal.pone.0227695)
Supplement: S3 Table — BFI scores range from 0–10, where low scores are good and high scores are bad. (DOCX) [file pone.0227695.s004.docx]

**S3 Table. Results from secondary outcome measures – BFI**

|  | **Randomised treatment** | | | | | | |  | | |
| --- | --- | --- | --- | --- | --- | --- | --- | --- | --- | --- |
|  | **PUFA** | | |  | **Olive Oil** | | |  |  |  |
|  | **N** | **Mean** | **SD** |  | **N** | **Mean** | **SD** | **Mean diff in change** | **95% CI** | **P-value** |
|  |  |  |  |  |  |  |  |  |  | **(t-test)** |
| **BFI (higher score = worse)** | | | | | | | | | | |
| Global fatigue baseline Score | 14 | 5.21 | 2.03 |  | 13 | 6.00 | 2.4 |  |  |  |
| Global fatigue week 8 score | 14 | 5.00 | 2.15 |  | 13 | 5.23 | 2.88 |  |  |  |
| Change from baseline (8 weeks -baseline) | 14 | -0.21 | 1.86 |  | 13 | -0.77 | 1.93 | 0.55 | (-0.95 ̶ 2.06) | 0.454 |

BFI scores range from 0-10, where low scores are good and high scores are bad.
