# Supplementary material for: A two-arm parallel double-blind randomised controlled pilot trial of the efficacy of Omega-3 polyunsaturated fatty acids for the treatment of women with endometriosis-associated pain (PurFECT1)
Source: PLoS One. 2020 Jan 17;15(1):e0227695. doi: 10.1371/journal.pone.0227695 (PMC6968860; doi:10.1371/journal.pone.0227695)
Supplement: S4 Table — GHQ scores range from 0–12, where higher scores represent higher levels of mental distress. (DOCX) [file pone.0227695.s005.docx]

**S4 Table. Results from secondary outcome measures – GHQ**

|  | **Randomised treatment** | | | | | | |  | | |
| --- | --- | --- | --- | --- | --- | --- | --- | --- | --- | --- |
|  | **PUFA** | | |  | **Olive Oil** | | |  |  |  |
|  | **N** | **Mean** | **SD** |  | **N** | **Mean** | **SD** | **Mean diff in change** | **95% CI** | **P-value** |
|  |  |  |  |  |  |  |  |  |  | **(t-test)** |
| **GHQ (higher score = worse)** | | | | | | | | | | |
| Global baseline score | 14 | 0.39 | 0.36 |  | 13 | 0.47 | 0.36 | - | - | - |
| Global week 8 score | 14 | 0.43 | 0.25 |  | 13 | 0.42 | 0.27 | - | - | - |
| Change from baseline (8 weeks -baseline) | 14 | 0.04 | 0.31 |  | 13 | -0.05 | 0.26 | 0.1 | (-0.13 ̶ 0.33) | 0.379 |

GHQ scores range from 0-12, where higher scores represent higher levels of mental distress.
