# Supplementary material for: A two-arm parallel double-blind randomised controlled pilot trial of the efficacy of Omega-3 polyunsaturated fatty acids for the treatment of women with endometriosis-associated pain (PurFECT1)
Source: PLoS One. 2020 Jan 17;15(1):e0227695. doi: 10.1371/journal.pone.0227695 (PMC6968860; doi:10.1371/journal.pone.0227695)
Supplement: S5 Table — WPAIQ scores range from 0–100, where low scores are good and high scores are bad indicating greater impairment and less productivity. (DOCX) [file pone.0227695.s006.docx]

**S5 Table. Results from secondary outcome measures – WPAIQ**

|  | **Randomised treatment** | | | | | | | |  | | |
| --- | --- | --- | --- | --- | --- | --- | --- | --- | --- | --- | --- |
|  | **PUFA** | | | |  | **Olive Oil** | | |  |  |  |
|  | **N** | | **Mean** | **SD** |  | **N** | **Mean** | **SD** | **Mean diff in change** | **95% CI** | **P-value** |
|  |  |  |  |  |  |  |  |  |  |  | **(t-test)** |
| **WPAIQ (higher score = worse)** | | | | | | | | | | | |
| Absenteeism_baseline_score | 9 | 2.15 | | 4.3 |  | 7 | 18.37 | 37.54 | - | - | - |
| Absenteeism_week_8_score | 9 | 14 | | 32.8 |  | 7 | 10.32 | 17.86 | - | - | - |
| Change from baseline (8 weeks -baseline) | 9 | 11.85 | | 33.11 |  | 7 | -8.05 | 42.17 | 19.89 | (-20.37 - 60.17) | 0.307 |
| Change from baseline (8 weeks -baseline) (absenteeism diff transformed) | 9 | 0.35 | | 0.68 |  | 7 | 0.66 | 0.86 | -0.31 | (-1.14 - 0.51) | 0.428 |
| Presenteesism_baseline_score | 9 | 35.6 | | 23.51 |  | 7 | 48.58 | 34.36 | - | - | - |
| Presenteesism_week_8_score | 9 | 46.7 | | 33.17 |  | 7 | 54.29 | 36.9 | - | - | - |
| Change from baseline (8 weeks -baseline) | 9 | 11.11 | | 32.19 |  | 7 | 5.71 | 51.59 | 5.39 | (-39.59 - 50.39) | 0.801 |
| Change from baseline (8 weeks -baseline) (presenteesism diff transformed) | 9 | 0.94 | | 0.62 |  | 7 | 1.43 | 0.38 | -0.49 | (-1.08 - 0.10) | 0.095 |
| Work_productivity_loss_baseline_score | 9 | -34.56 | | 23.51 |  | 7 | -47.58 | 34.36 | - | - | - |
| Work_productivity_loss_week_8_score | 9 | -45.67 | | 33.17 |  | 7 | -53.29 | 36.9 | - | - | - |
| Change from baseline (8 weeks -baseline) | 9 | -11.11 | | 32.19 |  | 7 | -5.71 | 51.59 | -5.39 | (-50.39 - 39.59) | 0.801 |
| Change from baseline (8 weeks -baseline) (work_productivity_loss diff_transformed) | 9 | 0.97 | | 0.64 |  | 7 | 1.42 | 0.39 | -0.45 | (-1.04 - 0.14) | 0.125 |
| Activity_impairment_baseline_score | 9 | 44.45 | | 26 |  | 7 | 42.86 | 23.6 | - | - | - |
| Activity_impairment_week_8_score | 9 | 48.89 | | 31.8 |  | 7 | 58.57 | 36.71 | - | - | - |
| Change from baseline (8 weeks-baseline) | 9 | 4.44 | | 27.43 |  | 7 | 15.71 | 37.79 | -11.26 | (-46.17 - 23.63) | 0.5 |
| Change from baseline (8 weeks-baseline) (activity_impairment diff_transformed) | 9 | 0.89 | | 0.71 |  | 7 | 1.34 | 0.61 | -0.44 | (-1.17 - 0.28) | 0.214 |

WPAIQ scores range from 0-100, where low scores are good and high scores are bad indicating greater impairment and less productivity.
