# Supplementary material for: A two-arm parallel double-blind randomised controlled pilot trial of the efficacy of Omega-3 polyunsaturated fatty acids for the treatment of women with endometriosis-associated pain (PurFECT1)
Source: PLoS One. 2020 Jan 17;15(1):e0227695. doi: 10.1371/journal.pone.0227695 (PMC6968860; doi:10.1371/journal.pone.0227695)
Supplement: S6 Table — 1 PCQ rumination scores range from 0–16, where low scores are good and high scores are bad. 2 PCQ magnification scores range from 0–12, where low scores are good and high scores are bad. 3 PCQ helplessness scores range from 0–24, where low scores are good and high scores are bad. 4 PCQ total scores range from 0–52, where low scores are good and high scores are bad. (DOCX) [file pone.0227695.s007.docx]

**S6 Table. Results from secondary outcome measures – PCQ**

|  | **Randomised treatment** | | | | | | |  | | |
| --- | --- | --- | --- | --- | --- | --- | --- | --- | --- | --- |
|  | **PUFA** | | |  | **Olive Oil** | | |  |  |  |
|  | **N** | **Mean** | **SD** |  | **N** | **Mean** | **SD** | **Mean diff in change** | **95% CI** | **P-value** |
|  |  |  |  |  |  |  |  |  |  | **(t-test)** |
| **PCQ (higher score = worse)** | | | | | | | | | | |
| Rumination baseline score^1^ | 14 | 1.92 | 1.13 |  | 13 | 2.44 | 1.06 | - | - | - |
| Rumination week 8 score | 14 | 1.54 | 0.85 |  | 13 | 1.52 | 1.06 | - | - | - |
| Change from baseline (8 weeks-baseline) | 14 | -0.38 | 0.67 |  | 13 | -0.92 | 1.1 | 0.54 | (-0.17 ̶ 1.26) | 0.128 |
| Magnification baseline score^2^ | 14 | 1.53 | 0.78 |  | 13 | 1.89 | 1.03 | - | - | - |
| Magnification week 8 score | 14 | 1.55 | 0.87 |  | 13 | 1.48 | 0.97 | - | - | - |
| Change from baseline (8 weeks -baseline) | 14 | 0.02 | 0.80 |  | 13 | -0.41 | 1.08 | 0.43 | (-0.32 ̶ 1.18) | 0.245 |
| Helplessness baseline score^3^ | 14 | 1.74 | 1.17 |  | 13 | 2.37 | 1.01 | - | - | - |
| Helplessness week 8 score | 14 | 1.79 | 0.70 |  | 13 | 1.52 | 0.94 | - | - | - |
| Change from baseline (8 weeks-baseline) | 14 | 0.05 | 0.85 |  | 13 | -0.85 | 1.15 | 0.89 | (0.1 ̶ 1.7) | 0.030 |
| Total baseline score^4^ | 14 | 1.72 | 0.90 |  | 13 | 2.24 | 0.93 | - | - | - |
| Total week 8 score | 14 | 1.62 | 0.69 |  | 13 | 1.51 | 0.94 | - | - | - |
| Change from baseline (8 weeks -baseline) | 14 | -0.10 | 0.58 |  | 13 | -0.73 | 1 | 0.63 | (- 0.02 ̶ 1.27) | 0.056 |

^1^ PCQ rumination scores range from 0-16, where low scores are good and high scores are bad. ^2^ PCQ magnification scores range from 0-12, where low scores are good and high scores are bad. ^3^ PCQ helplessness scores range from 0-24, where low scores are good and high scores are bad. ^4^ PCQ total scores range from 0-52, where low scores are good and high scores are bad.
