# Supplementary material for: A two-arm parallel double-blind randomised controlled pilot trial of the efficacy of Omega-3 polyunsaturated fatty acids for the treatment of women with endometriosis-associated pain (PurFECT1)
Source: PLoS One. 2020 Jan 17;15(1):e0227695. doi: 10.1371/journal.pone.0227695 (PMC6968860; doi:10.1371/journal.pone.0227695)
Supplement: S7 Table — 1 SAQ pleasure scores range from 0–18, where low scores are bad and high scores are good. 2 SAQ discomfort scores range from 0–6, where low scores are bad and high scores are good. 3 SAQ habit scores range from 0–3, where low scores are bad and high scores are good. 4 SAQ tired scores range from 0–3, where low scores are bad and high scores are good. (DOCX) [file pone.0227695.s008.docx]

**S7 Table. Results from secondary outcome measures – SAQ**

|  | **Randomised treatment** | | | | | | |  | | |
| --- | --- | --- | --- | --- | --- | --- | --- | --- | --- | --- |
|  | **PUFA** | | |  | **Olive Oil** | | |  |  |  |
|  | **N** | **Mean** | **SD** |  | **N** | **Mean** | **SD** | **Mean diff in change** | **95% CI** | **P-value** |
|  |  |  |  |  |  |  |  |  |  | **(t-test)** |
| **SAQ (higher score = better)** | | | | | | | | | | |
| Pleasure baseline score^1^ | 7 | 1.26 | 0.8 |  | 10 | 0.63 | 0.7 | - | - | - |
| Pleasure week 8 score | 7 | 1.19 | 1.03 |  | 10 | 0.73 | 0.81 | - | - | - |
| Change from baseline (8 weeks-baseline) | 7 | -0.07 | 0.83 |  | 10 | 0.1 | 0.95 | -0.17 | (-1.12 ̶ 0.78) | 0.705 |
| Discomfort baseline score^2^ | 7 | 0.95 | 0.61 |  | 10 | 1 | 1.22 | - | - | - |
| Discomfort week 8 score | 7 | 1.1 | 0.73 |  | 10 | 0.95 | 1.23 | - | - | - |
| Change from baseline (8 weeks -baseline) | 7 | 0.15 | 0.9 |  | 10 | -0.05 | 0.72 | 0.19 | (-0.65 ̶ 1.03) | 0.631 |
| Habit baseline score^3^ | 7 | 0.43 | 0.79 |  | 10 | 0.3 | 0.48 | - | - | - |
| Habit week 8 score | 7 | 0.43 | 0.79 |  | 10 | 0.4 | 0.7 | - | - | - |
| Change from baseline (8 weeks -baseline) | 7 | 0 | 0 |  | 10 | 0.1 | 0.74 | -0.1 | (-0.70 ̶ 0.50) | 0.728 |
| Tired baseline score^4^ | 7 | 1 | 1.41 |  | 10 | 1.8 | 1.23 | - | - | - |
| Tired week 8 score | 7 | 2 | 1 |  | 10 | 2.1 | 1.45 | - | - | - |

^1^ SAQ pleasure scores range from 0-18, where low scores are bad and high scores are good. ^2^ SAQ discomfort scores range from 0-6, where low scores are bad and high scores are good. ^3^ SAQ habit scores range from 0-3, where low scores are bad and high scores are good. ^4^ SAQ tired scores range from 0-3, where low scores are bad and high scores are good.
