# Supplementary material for: A two-arm parallel double-blind randomised controlled pilot trial of the efficacy of Omega-3 polyunsaturated fatty acids for the treatment of women with endometriosis-associated pain (PurFECT1)
Source: PLoS One. 2020 Jan 17;15(1):e0227695. doi: 10.1371/journal.pone.0227695 (PMC6968860; doi:10.1371/journal.pone.0227695)
Supplement: S8 Table — PDQ scores range from 0–35, where low scores are good and high scores are bad. (DOCX) [file pone.0227695.s009.docx]

**S8 Table. Results from secondary outcome measures – PDQ**

|  | **Randomised treatment** | | | | | | |  | | |
| --- | --- | --- | --- | --- | --- | --- | --- | --- | --- | --- |
|  | **PUFA** | | |  | **Olive Oil** | | |  |  |  |
|  | **N** | **Mean** | **SD** |  | **N** | **Mean** | **SD** | **Mean diff in change** | **95% CI** | **P-value** |
|  |  |  |  |  |  |  |  |  |  | **(t-test)** |
| **PDQ (higher score = worse)** | | | | | | | | | | |
| Global baseline score | 14 | 1.58 | 0.63 |  | 12 | 1.87 | 0.72 | - | - | - |
| Global week 8 score | 14 | 1.34 | 0.64 |  | 12 | 1.76 | 0.86 | - | - | - |
| Change from baseline (8 weeks -baseline) | 14 | -0.24 | 0.47 |  | 12 | -0.11 | 0.48 | -0.13 | (-0.51 ̶ 0.25) | 0.499 |

PDQ scores range from 0-35, where low scores are good and high scores are bad
