# Supplementary material for: A two-arm parallel double-blind randomised controlled pilot trial of the efficacy of Omega-3 polyunsaturated fatty acids for the treatment of women with endometriosis-associated pain (PurFECT1)
Source: PLoS One. 2020 Jan 17;15(1):e0227695. doi: 10.1371/journal.pone.0227695 (PMC6968860; doi:10.1371/journal.pone.0227695)
Supplement: S1 Protocol — (PDF) [file pone.0227695.s010.pdf]

Protocol

PURified Fatty acids for Endometriosis Clinical Trial 2

PurFECT

CI Prof Andrew Horne

Study Protocol

PURified Fatty acids for Endometriosis Clinical Trial

PurFECT

Co-sponsors	University of Edinburgh & NHS Lothian<br>ACCORD<br>The Queen's Medical Research Institute<br>47 Little France Crescent<br>Edinburgh<br>EH16 4TJ
Funder	University of Edinburgh
Chief Investigator	Prof Andrew W Horne
REC Number	16/SS/0010
Version Number and Date	Version 2_25th January 2016

COORDINATING CENTRE AND SITE

Chief Investigator

Professor Andrew W Horne *

MRC Centre for Reproductive Health
QMRI
47 Little France Crescent
Edinburgh
EH16 4TJ

Tel: 0131 242 6162
Fax: 0131 242 2686
Email: Andrew.horne@ed.ac.uk

Co-sponsor Representative

Ms Jo-Anne Robertson
Research Governance Coordinator
Research Governance & QA Office
University of Edinburgh
Doorway 6, School of Health and Social Sciences Teviot Place
Edinburgh
EH8 9AG

Tel:0131 651 5135
Email: Jo-Anne.Robertson@ed.ac.uk
www.accord.ed.ac.uk

NHS Lothian co-sponsor

Susan Shepherd
Head of Research Governance
The Queen's Medical Research Institute
47 Little France Crescent
Edinburgh, EH16 4TJ
Tel: 0131 242 3359
Fax: 0131 242 3343

Email:susan.shepherd@nhslothian.scot.nhs.uk

Trial Manager

Mrs Ann M Doust *
Room S7128 Simpson Centre for Reproductive Health
51 Little France Crescent
Edinburgh
EH16 4SA

Tel: 0131 242 9492
Fax: 0131 242 2686

Email: Ann.doust@ed.ac.uk

Trial Statistician

Dr. Tania Wood
9 Rowland Street
Toronto
M6P 1M2
Canada

Tel: 001 647 204 4443
Fax: 001 416 306 1450

Email: tania.wood88@gmail.com

Oxford Principal Investigator	Clinical research fellow
Associate Professor Christian Becker<br>Nuffield Department of Obstetrics and Gynaecology<br>University of Oxford<br>John Radcliffe Hospital - Women's Centre<br>Oxford<br>OX3 9DU<br>Tel: 01865 513866<br>Fax: 01865 221001<br>Email: christian.becker@obs-gyn.ox.ac.uk	Dr. Ibtisam Abokhrais *<br>Room S7.129 Simpson Centre for Reproductive Health<br>51 Little France Crescent<br>Edinburgh<br>EH16 4SA<br>Tel: 0131 242 2692<br>Fax: 0131 242 2686<br><br>Email: Ibtisam.Abokhrais@ed.ac.uk

*Protocol authors

CONTENTS

1 INTRODUCTION 9
1.1 BACKGROUND 9
1.2 RATIONALE FOR STUDY 9
2 STUDY OBJECTIVES 9
2.1 OBJECTIVES 9
2.1.1 Primary Objective 9
2.1.2 Secondary Objectives 10
2.2 ENDPOINTS 10
2.2.1 Primary Endpoint 10
2.2.2 Secondary Endpoints 10
3 STUDY DESIGN 10
4 STUDY POPULATION 10
4.1 NUMBER OF PARTICIPANTS 10
4.2 INCLUSION CRITERIA 11
4.3 EXCLUSION CRITERIA 11
5 PARTICIPANT SELECTION AND ENROLMENT 11
5.1 IDENTIFYING PARTICIPANTS 11
5.2 CONSENTING PARTICIPANTS 11
5.3 SCREENING FOR ELIGIBILITY 12
5.4 INELIGIBLE AND NON-RECRUITED PARTICIPANTS 12
5.5 RANDOMISATION 12
5.5.1 Randomisation Procedures 12
5.5.2 Treatment Allocation 12
5.5.3 Emergency Unblinding Procedures 12
5.5.4 Withdrawal of Study Participants 12
6 INVESTIGATIONAL MEDICINAL PRODUCT AND PLACEBO 13
6.1 STUDY DRUG .... Error! Bookmark not defined.
6.1.1 Study Drug Identification 13
6.1.2 Study Drug Manufacturer 13
6.1.3 Marketing Authorisation Holder 13
6.1.4 Labelling and Packaging 13
6.1.5 Storage 13
6.1.6 Summary of Product Characteristics or Investigators Brochure 14
6.2 PLACEBO .... Error! Bookmark not defined.
6.3 DOSING REGIME 14
6.4 DOSE CHANGES 14
6.5 PARTICIPANT COMPLIANCE 14
6.6 OVERDOSE 14
6.7 OTHER MEDICATIONS 14
6.7.1 Non-Investigational Medicinal Products 14

6.7.2 Permitted Medications ... 14
6.7.3 Prohibited Medications ... 14
7 STUDY ASSESSMENTS ... 14
7.1 SAFETY ASSESSMENTS ... 14
7.2 STUDY ASSESSMENTS ... 15
8 DATA COLLECTION ... 15
9 STATISTICS AND DATA ANALYSIS ... 17
9.1 SAMPLE SIZE CALCULATION ... 17
9.2 PROPOSED ANALYSES ... 17
10 ADVERSE EVENTS ... 18
10.1 DEFINITIONS ... 18
10.2 IDENTIFYING AEs AND SAEs ... 19
10.3 RECORDING AEs AND SAEs ... 19
10.4 ASSESSMENT OF AEs AND SAEs ... 19
10.4.1 Assessment of Seriousness ... 19
10.4.2 Assessment of Causality ... 19
10.4.3 Assessment of Expectedness ... 20
10.4.4 Assessment of Severity ... 20
10.5 REPORTING OF SAEs/SARs/SUSARs ... 20
10.6 REGULATORY REPORTING REQUIREMENTS ... 21
10.7 FOLLOW UP PROCEDURES ... 21
11 PREGNANCY ... 21
12 TRIAL MANAGEMENT AND OVERSIGHT ARRANGEMENTS ... 22
12.1 TRIAL MANAGEMENT GROUP ... 22
12.2 TRIAL STEERING COMMITTEE ... 22
12.3 DATA MONITORING COMMITTEE ... 22
12.4 INSPECTION OF RECORDS ... 22
12.5 RISK ASSESSMENT ... 22
12.6 STUDY MONITORING AND AUDIT ... 22
13 GOOD CLINICAL PRACTICE ... 23
13.1 ETHICAL CONDUCT ... 23
13.2 REGULATORY COMPLIANCE ... 23
13.3 INVESTIGATOR RESPONSIBILITIES ... 23
13.3.1 Informed Consent ... 23
13.3.2 Study Site Staff ... 23
13.3.3 Data Recording ... 24
13.3.4 Investigator Documentation ... 24
13.3.5 GCP Training ... 24
13.3.6 Confidentiality ... 24
13.3.7 Data Protection ... 24
14 STUDY CONDUCT RESPONSIBILITIES ... 25
14.1 PROTOCOL AMENDMENTS ... 25

14.2 PROTOCOL VIOLATIONS AND DEVIATIONS 25
14.3 SERIOUS BREACH REQUIREMENTS 25
14.4 STUDY RECORD RETENTION 25
14.5 END OF STUDY 25
14.6 CONTINUATION OF DRUG FOLLOWING THE END OF STUDY 26
14.7 INSURANCE AND INDEMNITY 26

15 REPORTING, PUBLICATIONS AND NOTIFICATION OF RESULTS 27

15.1 AUTHORSHIP POLICY 27
15.2 PUBLICATION 27
15.3 PEER REVIEW 27

16 References..

LIST OF ABBREVIATIONS

ACCORD	Academic and Clinical Central Office for Research & Development - Joint office for University of Edinburgh and NHS Lothian
AE	Adverse Event
AR	Adverse Reaction
BFI	Brief Fatigue Inventory
BPI	Brief Pain Inventory
CRF	Case Report Form
GCP	Good Clinical Practice
ICH	International Conference on Harmonisation
ISF	Investigator Site File
ISG	Investigational Supplies Group
N/A	Not applicable
NRS	Numerical Rating Scale
PUFA	Purified fatty acids
RCT	Randomised Control Trial
SAE	Serious Adverse Event
SAR	Serious Adverse Reaction
SOP	Standard Operating Procedure
SUSAR	Suspected Unexpected Serious Adverse Reaction
TMF	Trial Master File
UAR	Unexpected Adverse Reaction

SUMMARY

Endometriosis (womb lining outside the womb) affects 6-10% of women and is associated with debilitating chronic pelvic pain. It costs the UK >£2.8 billion per year in loss of productivity. Endometriosis is managed surgically or medically. However, ~75% symptoms recur after surgery and available medical treatments ('anti-hormones') have undesirable side effects and are contraceptive. Omega-3 purified fatty acids (PUFA) have been shown in animal models to reduce factors that are thought to lead to endometriosis-associated pain, have minimal side effects and no effects on fertility. We plan to perform a feasibility study to inform planning of a future multicentre trial to evaluate the efficacy of PUFA in the management of endometriosis-associated pain.

INTRODUCTION

1.1 BACKGROUND

Endometriosis is a common, chronic inflammatory disease, affecting 6-10% of women of reproductive age[1]. Endometriosis is defined by endometrial tissue implanted in ectopic sites, usually within the peritoneal cavity. It is associated with chronic pelvic pain, pain during menstruation, pain during intercourse and an increased likelihood of infertility[2]. Endometriosis is managed surgically or medically. However, ~75% symptoms recur after surgery and available medical treatments ('anti-hormones') can cause menopause-like symptoms and are contraceptive[3]. There exists an unmet clinical need for better treatments for endometriosis with few or minimal side effects and that have no effect on fertility.

1.2 RATIONALE FOR STUDY

We are exploring a new treatment strategy involving highly purified Omega-3 fatty acids (PUFA).

PUFA are a dietary supplement and have been shown in in-vitro laboratory and animal models to reduce factors that lead to endometriosis-associated pain. In an in-vitro laboratory study, it has been demonstrated that specific PUFA ratios have a suppressive effect on the survival of endometrial cells[4.] In a study using a rat model of endometriosis, specific PUFA ratios have been shown to reduce the inflammation associated with endometriotic lesions and suppress progression of the disease[5].

To determine whether PUFA will reduce endometriosis-associated pain requires a large multicentre RCT. We propose a two centre feasibility study to inform the planning of this future RCT.

2 STUDY OBJECTIVES

2.1 OBJECTIVES

2.1.1 Primary Objective

To determine whether it is possible to achieve acceptable recruitment and retention rates within defined inclusion/exclusion criteria.

2.1.2 Secondary Objectives

To determine the acceptability to patients of the proposed methods of recruitment, randomisation and treatments.

2.2 ENDPOINTS

2.2.1 Primary Endpoint

1. The proportion of screened women who are eligible for the trial.
2. The proportion of eligible patients randomised into the study.
3. The proportion of randomised patients who take the supplement and complete questionnaires at final follow up.

2.2.2 Secondary Endpoints

The remaining data (effectiveness and acceptability of proposed methods of recruitment, randomisation, treatments and questionnaires to study the effect of PUFA in the management of endometriosis-associated pelvic pain) will be used to refine the research methodology of a future large RCT.

3 STUDY DESIGN

A two-arm parallel randomised controlled pilot feasibility study.

We will aim to recruit as many participants as possible within 12 months with the goal of reaching at least 60 (max 100) between two centres in Edinburgh and Oxford. Eligibility will be determined after monitoring pain scores over a 4 week period. If eligible, participants will be randomised to PUFA or placebo, and receive treatment for 8 weeks. Pain scores will be monitored over the last 4 weeks, score of 4 on at least 2 occasions is required. Unblinding will take place at the end of the study when all participants have completed the treatment phase.

4 STUDY POPULATION

4.1 NUMBER OF PARTICIPANTS

Women (aged 18-50) with a known diagnosis of endometriosis and associated pelvic pain of at least 3 months duration will be recruited from gynaecology outpatient departments in NHS Lothian and NHS Oxford Trust. We will recruit as many women as possible within a 12 month period.

4.2 INCLUSION CRITERIA

Aged 18-50 years

Known endometriosis diagnosed from previous laparoscopy (< 3 years ago)

Pain of at least 3 months duration located in the true pelvis

Worst pain score ≥4 over 4 weeks as measured by Numerical Rating Score (NRS)

Ability to provide informed consent

4.3 EXCLUSION CRITERIA

Unable to take/allergic to fish / PUFA / peanuts / soyabean

Insulin dependent diabetes

Pregnant

Taking contraindicated medications (anticoagulants)

4.4 CO-ENROLMENT

Other studies eg Pre-empt look at treatment of endometriosis using routine clinical methods. Participant can take part in both studies as there is no cross-over of treatment. One is for the treatment of endometriosis and this one looking at pain.

5 PARTICIPANT SELECTION AND ENROLMENT

5.1 IDENTIFYING PARTICIPANTS

Women will be identified by their clinician and asked if they will talk to a member of the clinical research team. Recruitment may be in a clinic environment and patients will only be approached once permission has been given by the participant to their direct clinical care team.

5.2 CONSENTING PARTICIPANTS

If they agree, they will then be given a patient information sheet and will be asked if they are happy to be contacted to get their decision on whether they wish to participate. The patient will be given the option of the research team contacting them or will be given the team's number to contact if interested in participating. This will give them the minimum of an hour to read the patient information sheet, ask any questions and make an informed choice about whether to participate or not. This might be after a clinic appointment so patients may be asked to go away and consider the information and come back on the same day. Participants will be consented by a member of the

clinical research team. Consent will only be taken by GCP trained members of staff who are on the delegation log.

5.3 SCREENING FOR ELIGIBILITY

If participants express an interest in the study they will be assessed to determine whether they fulfil all the potential eligibility criteria. Eligibility for treatment will be based on the worst of the 4 weekly NRS. A worst pain score (NRS) of greater than or equal to (≥) 4 is required for randomisation to treatment.

5.4 INELIGIBLE AND NON-RECRUITED PARTICIPANTS

The following information will be monitored and collected:

- Demographic information (age, initials, date of clinic appointment)
- Reason for not entering the randomisation phase (if available and offered)

5.5 RANDOMISATION

5.5.1 Randomisation Procedures

Eligible women will be randomised to either PUFA or comparator. The randomisation scheme will be drawn up by an independent person who is not involved in the study and the allocated treatment codes will be put into sealed, consecutively numbered opaque envelopes. When a patient is randomised into the study, she will receive the treatment indicated in the next available envelope. These will be kept with the trial manager in a secure office.

5.5.2 Treatment Allocation

After randomisation, participants will be allocated an oral treatment pack. The participants will be allowed to use other medication (including analgesics) throughout the study period. These concomitant medications will be recorded.

The treatment allocated is a licensed supplement (Omacor).

5.5.3 Emergency Unblinding Procedures

The blinding code will only be broken in emergency situations for reasons of patient safety, where knowledge of the treatment administered is necessary for the treatment of a serious adverse event or when required by local regulatory authorities. Participants whose randomisation codes are broken will cease treatment, but will continue to be followed up. The Investigator must document the reason for breaking the blind in the participant's source documents and in the Investigator Site Master File. An unblinding envelope will be in the pharmacy file and one will be held by the sponsor for unblinding in the event of a SUSAR.

5.5.4 Withdrawal of Study Participants

Participants may discontinue from the trial at any time at their own request, or they may be withdrawn at the discretion of the Investigator or sponsor for

safety, behavioural or administrative reasons. Withdrawal may be from treatment or from the whole trial.

Withdrawal from treatment

If a participant wishes to withdraw from treatment but is willing to complete follow-up she will be asked to return for a final visit to complete questionnaires and any adverse events will be followed up to resolution.

Withdrawal of consent

If a participant wishes to withdraw consent the investigator will inquire about the reason for withdrawal and request that the subject return all unused supplements. With permission all data obtained up to point of withdrawal will be used.

Loss to follow up

If a participant does not return for a scheduled visit, every effort will be made to contact the participant. All data obtained up to point of loss to follow-up will be used.

6 SUPPLEMENT AND COMPARATOR

6.1 Supplement

6.1.1 Supplement Identification

High dose purified fatty acid

6.1.2 Supplement Manufacturer

ISG

6.1.3 Marketing Authorisation Holder

Not applicable

6.1.4 Labelling and Packaging

Investigational Supplies Group (ISG)

1 George Square, Edinburgh, EH8 9JZ

6.1.5 Storage

Below 25°C

6.1.6 Summary of Product Characteristics or Investigators Brochure

Not applicable

6.2 Comparator

Olive oil softgelatin capsules from EuroCaps

6.3 DOSING REGIME

Two capsules per day for 8 weeks.

6.4 DOSE CHANGES

There will be no dose changes throughout the course of the study.

6.5 PARTICIPANT COMPLIANCE

Compliance will be measured via a treatment diary, phone call at week 6-8 and serum levels at end of treatment.

6.6 OVERDOSE

There are no special recommendations.

6.7 OTHER MEDICATIONS

6.7.1 Non-Investigational Medicinal Products

N/A

6.7.2 Permitted Medications

No restrictions.

6.7.3 Prohibited Medications

None. Precautions should be taken when taken in conjunction with anti-coagulants.

7 STUDY ASSESSMENTS

7.1 SAFETY ASSESSMENTS

- Medical history
- Pregnancy test
- Adverse events

7.2 STUDY ASSESSMENTS

(-4 weeks): **Visit 1**

Informed consent
Screening for eligibility, past medical history, BMI, Medication use
Eligibility
Pain score (NRS) = average and worst score over previous week

Pain assessment

Week -3 Pain score (NRS)
Week -2 Pain score (NRS)
Week-1 Pain score (NRS)

Week 0 (Baseline) **Visit 2**

Eligibility confirmed∗
Randomisation and treatment allocation
Treatment diary
Questionnaires
Blood sample
Pregnancy test

Week 0-8 **Treatment phase**

Pain assessment

Week 5 Pain score (NRS)
Week 6 Pain score (NRS)
Week 7 Pain score (NRS)

Week 8 **Visit 3**

Pain score (NRS)
Adverse events
Questionnaires
Collection of treatment diary
Collection of any unused study medications
Blood sample

∗Based on worst NRS score ≥4. If ineligible at this point – thanked for participation.

8 DATA COLLECTION

Screening: A member of the research team will carry out a screening visit to assess eligibility. All data will be recorded on a CRF and transferred to a secure database.

Participant Log: The clinical research team will keep an electronic log of women who fulfil the eligibility criteria, women who are invited to participate in the study, women recruited and women who leave the trial early. Reasons for non-recruitment (eg non-eligibility, refusal to participate, administrative error) will also be recorded. We will attempt to collect reasons for non-participation from women who decline to take part after previously providing contact details. We will ask, with permission the reason for not wanting to participate as this will give us useful information on the design of future studies. During the course of the study, we will document reasons for withdrawal from the study and loss to follow-up.

Treatment diaries: details are documented in Section 6.5.

Questionnaires: A questionnaire will be given to all participants at randomisation and on trial completion (baseline and 12 weeks). This will include questions to capture the baseline demographic and clinical characteristics of the participants and include the following validated tools:

Numerical rating score (NRS)

SF-12 quality of life

Brief Pain Inventory (BPI)

Pain catastrophising (PCQ)

PainDETECT™

Sexual Activity Questionnaires (SAQ)

Brief Fatigue Inventory (BFI)

General Health Questionnaire -12 (GHQ-12)

Work and Productivity Activity Impairment Questionnaire- Specific Health Problem version 2.0 (WPAI-SHP)

Acceptability questionnaire (End of study only)

We are collecting multiple questionnaires to assess which ones give us the best outcomes for a future randomised controlled study and which ones are completed fully. The pain questionnaires all ask about different aspects of pain eg neuropathic so we would like to know how acceptable the completion is as there can be some repetition.

Blood samples: The plasma fraction from 5 ml of peripheral venous blood sample will be collected and processed for EPA, AA, and DHA analysis. Analysis will take place in the University of Stirling, Institute of Aquaculture. Samples may be retained for use in future ethically approved projects. These will be stored in the MRC Centre for Reproductive Health laboratory in the QMRI. All samples will be anonymised before analysis.

9 STATISTICS AND DATA ANALYSIS

9.1 SAMPLE SIZE CALCULATION

The emphasis in this study is to establish feasibility, not statistical significance. This study is designed primarily to explore the effectiveness of the proposed field methodology: recruitment, retention and compliance. We will aim to recruit as many women as possible over a 12 month period. We estimate that we will recruit ~ 4-5 patients per month and will aim to recruit 60 patients. Data from this pilot study will be used to refine sample size calculations for any future RCT.

9.2 PROPOSED ANALYSES

Primary objective

Determine recruitment and retention rates. Using the information collected from the participant log, we will determine the number of patients recruited from the pool of eligible women and a >50% recruitment will be deemed acceptable. While a retention rate of 100% would be ideal, we will consider a rate of 80% satisfactory. We will provide an estimate of the proportion and its 95% confidence interval. In addition, we will determine the nature and number of unanswered questions in each questionnaire. We aim to determine whether the trial design will perform well enough in the field to warrant rolling out the study to full trial. All analysis will be carried out in the University of Edinburgh.

Secondary objectives

Acceptability of proposed methods of recruitment, randomisation, treatments, and questionnaires to study the effect of PUFA in the management of endometriosis-associated pelvic pain will be assessed quantitatively using acceptability questionnaires at end of study. Due to the conflicting literature about the benefits of methods such as prescription monitoring, pill counting and devices for monitoring the self-administration of medicines, data on blinding and compliance to treatment will also be derived from these questionnaires. We also aim to determine if treatment is acceptable in terms of self-reported compliance (from treatment diaries). Although this is a pilot study and the sample size is small, we will assess the effect of any non-compliance on the LICKERT score by performing protocol and intention-to-treat analyses. This information will be used to inform the design of the future RCT.

10 ADVERSE EVENTS

The Investigator is responsible for the detection and documentation of events meeting the criteria and definitions detailed below.

Participants will be instructed to contact their Investigator at any time after consenting to join the trial if any symptoms develop. All adverse events (AE) that occur after joining the trial must be reported in detail in the Case Report Form (CRF) or AE form. In the case of an AE, the Investigator should initiate the appropriate treatment according to their medical judgment. Participants with AEs present at the last visit must be followed up until resolution of the event.

10.1 DEFINITIONS

An **adverse event** (AE) is any untoward medical occurrence in a clinical trial participant which does not necessarily have a causal relationship with a Supplement.

An **adverse reaction** (AR) is any untoward and unintended response to a Supplement which is related to any dose administered to that participant.

A **serious adverse event** (SAE), **serious adverse reaction** (SAR). Any AE or AR that at any dose:

- results in death of the clinical trial participant;
- is life threatening*;
- requires in-patient hospitalisation^ or prolongation of existing hospitalisation;
- results in persistent or significant disability or incapacity;
- consists of a congenital anomaly or birth defect;
- results in any other significant medical event not meeting the criteria above.

*Life-threatening in the definition of an SAE or SAR refers to an event where the participant was at risk of death at the time of the event. It does not refer to an event which hypothetically might have caused death if it were more severe.

^Any hospitalisation that was planned prior to randomisation will not meet SAE criteria. Any hospitalisation that is planned post randomisation will meet the SAE criteria.

Hospitalisation for a pre-existing condition eg exacerbation of pain due to endometriosis will not be considered a SAE but will be documented.

A suspected unexpected serious adverse reaction (SUSAR) is any AR that is classified as serious and is suspected to be caused by the IMP, that it is not consistent with the information about the IMP in the Summary of Product Characteristics (SmPC) or Investigators Brochure. **As this is a supplement and not an IMP this is not anticipated**.

10.2 IDENTIFYING AEs AND SAEs

All AEs and SAEs will be recorded from the time a participant signs the consent form to take part in the study until end of treatment.

Participants will be asked about the occurrence of AEs/SAEs at every visit during the study. Open-ended and non-leading verbal questioning of the participant will be used to enquire about AE/SAE occurrence. Participants will also be asked if they have been admitted to hospital, had any accidents, used any new medicines or changed concomitant medication regimens. If there is any doubt as to whether a clinical observation is an AE, the event will be recorded.

Common ailments, e.g. coughs and colds, will not be recorded as AEs.

AEs and SAEs may also be identified via information from support departments e.g. laboratories.

10.3 RECORDING AEs AND SAEs

When an AE/SAE occurs, it is the responsibility of the Investigator to review all documentation (e.g. hospital notes, laboratory and diagnostic reports) related to the event. The Investigator will then record all relevant information in the CRF and on the SAE form (if the AE meets the criteria of serious).

Information to be collected includes dose, type of event, onset date, Investigator assessment of severity and causality, date of resolution as well as treatment required, investigations needed and outcome.

10.4 ASSESSMENT OF AEs AND SAEs

Seriousness, causality, severity and expectedness will be assessed by the Principal Investigator. For randomised studies, AEs will be assessed as though the participant is taking supplements. Cases that are considered serious, possibly, probably or definitely related to the supplement and unexpected (i.e. SUSARs) will be unblinded.

The Investigator is responsible for assessing each AE.

The Chief Investigator (CI) may not downgrade an event that has been assessed by an Investigator as an SAE or SUSAR, but can upgrade an AE to an SAE, SAR or SUSAR if appropriate.

10.4.1 Assessment of Seriousness

The Investigator will make an assessment of seriousness as defined in Section 10.1.

10.4.2 Assessment of Causality

The Investigator will make an assessment of whether the AE/SAE is likely to be related to the IMP according to the definitions below.

- Unrelated: where an event is not considered to be related to the supplement.

- Possibly Related: The nature of the event, the underlying medical condition, concomitant medication or temporal relationship make it possible that the AE has a causal relationship to the study drug. The assessment of causality will be made against the reference safety information found in the

Where non Investigational Medicinal Products (NIMPs) e.g. rescue/escape drugs are given: if the AE is considered to be related to an interaction between the supplement and the NIMP, or where the AE might be linked to either the IMP or the NIMP but cannot be clearly attributed to either one of these, the event will be considered as an AR. Alternative causes such as natural history of the underlying disease, other risk factors and the temporal relationship of the event to the treatment should be considered and investigated. The blind should not be broken for the purpose of making this assessment.

10.4.3 Assessment of Expectedness

If an event is judged to be an AR, the evaluation of expectedness will be made based on knowledge of the reaction and the relevant product information documented

10.4.4 Assessment of Severity

The Investigator will make an assessment of severity for each AE/SAE and record this on the CRF or SAE form according to one of the following categories:

Mild: an event that is easily tolerated by the participant, causing minimal discomfort and not interfering with every day activities.

Moderate: an event that is sufficiently discomforting to interfere with normal everyday activities.

Severe: an event that prevents normal everyday activities.

Note: the term ‘severe’, used to describe the intensity, should not be confused with ‘serious’ which is a regulatory definition based on participant/event outcome or action criteria. For example, a headache may be severe but not serious, while a minor stroke is serious but may not be severe.

10.5 REPORTING OF SAEs/SARs/SUSARs

Once the Investigator becomes aware that an SAE has occurred in a study participant, the information will be reported to the ACCORD Research Governance & QA Office **immediately or within 24 hours**. If the Investigator does not have all information regarding an SAE, they should not wait for this additional information before notifying ACCORD. The SAE report form can be updated when the additional information is received.

The SAE report will provide an assessment of causality and expectedness at the time of the initial report to ACCORD according to Sections 10.4.2, Assessment of Causality and 10.4.3, Assessment of Expectedness.

The SAE form will be transmitted by fax to ACCORD on **+44 (0)131 242 9447** or may be transmitted by hand to the office or submitted via email to Safety.Accord@ed.ac.uk. Only forms in a pdf format will be accepted by ACCORD via email.

Where missing information has not been sent to ACCORD after an initial report, ACCORD will contact the investigator and request the missing information.

All reports faxed to ACCORD and any follow up information will be retained by the Investigator in the Investigator Site File (ISF).

10.6 REGULATORY REPORTING REQUIREMENTS

The ACCORD Research Governance & QA Office is responsible for pharmacovigilance reporting on behalf of the co-sponsors (Edinburgh University and NHS Lothian).

The ACCORD Research Governance & QA Office has a legal responsibility to notify the relevant ethics committee (Research Ethics Committee (REC) that approved the trial). Fatal or life threatening SUSARs will be reported no later than 7 calendar days and all other SUSARs will be reported no later than 15 calendar days after ACCORD is first aware of the reaction.

ACCORD will inform Investigators at participating sites of all SUSARs and any other arising safety information.

10.7 FOLLOW UP PROCEDURES

After initially recording an AE or recording and reporting an SAE, the Investigator will follow each participant until resolution or death of the participant. Follow up information on an SAE will be reported to the ACCORD office.

AEs still present in participants at the last study visit will be monitored until resolution of the event or until no longer medically indicated.

11 PREGNANCY

Pregnancy is not considered an AE or SAE; however, the Investigator will collect pregnancy information for any female participants who become pregnant while participating in the study. The Investigator will record the information on a Pregnancy Notification Form and submit this to the ACCORD office within 14 days of being made aware of the pregnancy.

All pregnant female participants will be followed up until the outcome of the pregnancy.

12 TRIAL MANAGEMENT AND OVERSIGHT ARRANGEMENTS

12.1 TRIAL MANAGEMENT GROUP

The trial will be coordinated by a Project Management Group, consisting of Chief Investigator, the Trial Manager and clinical research fellow.

The Trial Manager will oversee the study and will be accountable to the Chief Investigator. The Trial Manager will be responsible for checking the CRFs for completeness, plausibility and consistency. Any queries will be resolved by the Investigator or delegated member of the trial team.

A Delegation Log will be prepared for each site, detailing the responsibilities of each member of staff working on the trial.

12.2 TRIAL STEERING COMMITTEE

N/A

12.3 DATA MONITORING COMMITTEE

N/A

12.4 INSPECTION OF RECORDS

Investigators and institutions involved in the study will permit trial related monitoring and audits on behalf of the sponsor, REC review, and regulatory inspection(s). In the event of an audit or monitoring, the Investigator agrees to allow the representatives of the sponsor direct access to all study records and source documentation. In the event of regulatory inspection, the Investigator agrees to allow inspectors direct access to all study records and source documentation.

12.5 RISK ASSESSMENT

An independent risk assessment will be performed by an ACCORD Clinical Trials Monitor to determine if monitoring is required and if so, at what level. An independent risk assessment will also be carried out by the ACCORD Quality Assurance Group to determine if an audit should be performed before/during/after the study and if so, at what locations and at what frequency.

12.6 STUDY MONITORING AND AUDIT

An ACCORD Clinical Trials Monitor or an appointed monitor will visit the Investigator site prior to the start of the study and during the course of the study if required, in accordance with the monitoring plan if required. Risk assessment will determine if audit, by the ACCORD QA group, is required. Details will be captured in an audit plan. Audit of Investigator sites, study management activities and study collaborative units, facilities and 3rd parties may be performed.

13 GOOD CLINICAL PRACTICE

13.1 ETHICAL CONDUCT

The study will be conducted in accordance with the principles of the International Conference on Harmonisation Tripartite Guideline for Good Clinical Practice (ICH GCP).

A favorable ethical opinion will be obtained from the appropriate REC and local R&D approval will be obtained prior to commencement of the study.

13.2 COMPLIANCE

13.3 INVESTIGATOR RESPONSIBILITIES

The Investigator is responsible for the overall conduct of the study at the site and compliance with the protocol and any protocol amendments. In accordance with the principles of ICH GCP, the following areas listed in this section are also the responsibility of the Investigator. Responsibilities may be delegated to an appropriate member of study site staff.

13.3.1 Informed Consent

The Investigator is responsible for ensuring informed consent is obtained before any protocol specific procedures are carried out. The decision of a participant to participate in clinical research is voluntary and should be based on a clear understanding of what is involved.

Participants must receive adequate oral and written information – appropriate Participant Information and Informed Consent Forms will be provided. The oral explanation to the participant will be performed by the Investigator or qualified delegated person, and must cover all the elements specified in the Participant Information Sheet and Consent Form.

The participant must be given every opportunity to clarify any points they do not understand and, if necessary, ask for more information. The participant must be given sufficient time to consider the information provided. It should be emphasised that the participant may withdraw their consent to participate at any time without loss of benefits to which they otherwise would be entitled.

The participant will be informed and agree to their medical records being inspected by regulatory authorities and representatives of the sponsor(s) but understand that their name will not be disclosed outside the hospital.

The Investigator or delegated member of the trial team and the participant will sign and date the Informed Consent Form(s) to confirm that consent has been obtained. The participant will receive a copy of this document and a copy filed in the Investigator Site File (ISF) and participant's medical notes.

13.3.2 Study Site Staff

The Investigator must be familiar with the supplement, protocol and the study requirements. It is the Investigator's responsibility to ensure that all staff

assisting with the study are adequately informed about the supplement, protocol and their trial related duties.

13.3.3 Data Recording

The Principle Investigator is responsible for the quality of the data recorded in the CRF at each Investigator Site. The source data plan identifies which source data correspond to CRF data and states which data are recorded directly into the CRF.

13.3.4 Investigator Documentation

Prior to beginning the study, each Investigator will be asked to provide particular essential documents to the ACCORD Research Governance & QA Office, including but not limited to:

- Curriculum vitae (CV) signed and dated by the Investigator indicating that it is accurate and current.

The ACCORD Research Governance & QA Office will ensure all other documents required by ICH GCP are retained in a Trial Master File (TMF), where required, and that appropriate documentation is available in local ISFs.

13.3.5 GCP Training

All study staff must hold evidence of appropriate GCP training.

13.3.6 Confidentiality

All laboratory specimens, evaluation forms, reports, and other records must be identified in a manner designed to maintain participant confidentiality. All records must be kept in a secure storage area with limited access. Clinical information will not be released without the written permission of the participant. The Investigator and study site staff involved with this study may not disclose or use for any purpose other than performance of the study, any data, record, or other unpublished, confidential information disclosed to those individuals for the purpose of the study. Prior written agreement from the sponsor or its designee must be obtained for the disclosure of any said confidential information to other parties.

13.3.7 Data Protection

All Investigators and study site staff involved with this study must comply with the requirements of the Data Protection Act 1998 with regard to the collection, storage, processing and disclosure of personal information and will uphold the Act’s core principles. Access to collated participant data will be restricted to those clinicians treating the participants, representatives of the sponsor(s) and representatives of regulatory authorities.

Computers used to collate the data will have limited access measures via user names and passwords.

Published results will not contain any personal data that could allow identification of individual participants.

14 STUDY CONDUCT RESPONSIBILITIES

14.1 PROTOCOL AMENDMENTS

Any changes in research activity, except those necessary to remove an apparent, immediate hazard to the participant in the case of an urgent safety measure, must be reviewed and approved by the Chief Investigator.

Amendments to the protocol must be submitted in writing to the appropriate REC, and local R&D for approval prior to participants being enrolled into an amended protocol.

14.2 PROTOCOL VIOLATIONS AND DEVIATIONS

Prospective protocol deviations, i.e. protocol waivers, will not be approved by the sponsors and therefore will not be implemented, except where necessary to eliminate an immediate hazard to study participants. If this necessitates a subsequent protocol amendment, this should be submitted to the REC, and local R&D for review and approval if appropriate.

Protocol deviations will be recorded in a protocol deviation log and logs will be submitted to the sponsors every 3 months. Each protocol violation will be reported to the sponsor within 24 hours of becoming aware of the violation.

14.3 SERIOUS BREACH REQUIREMENTS

A serious breach is a breach which is likely to effect to a significant degree:

(a) The safety or physical or mental integrity of the participants of the trial; or

(b) The scientific value of the trial.

If a potential serious breach is identified by the Chief investigator, Principal Investigator or delegates, the co-sponsors (accord.seriousbreach@ed.ac.uk) must be notified within 24 hours. It is the responsibility of the co-sponsors to assess the impact of the breach on the scientific value of the trial, to determine whether the incident constitutes a serious breach and report to regulatory authorities and research ethics committees as necessary.

14.4 STUDY RECORD RETENTION

All study documentation will be kept for a minimum of 5 years from the protocol defined end of study point. When the minimum retention period has elapsed, study documentation will not be destroyed without permission from the sponsor.

14.5 END OF STUDY

The end of study is defined as the last participant's last visit.

The Investigators and/or the co-sponsor(s) have the right at any time to terminate the study for clinical or administrative reasons.

The end of the study will be reported to the REC and within 90 days, or 15 days if the study is terminated prematurely. The Investigators will inform participants of the premature study closure and ensure that the appropriate follow up is arranged for all participants involved.

A summary report of the study will be provided to the REC and within 1 year of the end of the study.

14.6 CONTINUATION OF DRUG FOLLOWING THE END OF STUDY

Participants will be unblinded at the end of the study and if they wish to continue treatment these are available across the counter at any pharmacy/health food shops.

14.7 INSURANCE AND INDEMNITY

The co-sponsors are responsible for ensuring proper provision has been made for insurance or indemnity to cover their liability and the liability of the Chief Investigator and staff.

The following arrangements are in place to fulfil the co-sponsors' responsibilities:

- The Protocol has been designed by the Chief Investigator and researchers employed by the University and collaborators. The University has insurance in place (which includes no-fault compensation) for negligent harm caused by poor protocol design by the Chief Investigator and researchers employed by the University.
- Sites participating in the study will be liable for clinical negligence and other negligent harm to individuals taking part in the study and covered by the duty of care owed to them by the sites concerned. The co-sponsors require individual sites participating in the study to arrange for their own insurance or indemnity in respect of these liabilities.
- Sites which are part of the United Kingdom's Nation Health Service will have the benefit of NHS Indemnity.
- Sites out with the United Kingdom will be responsible for arranging their own indemnity or insurance for their participation in the study, as well as for compliance with local law applicable to their participation in the study.
- The manufacturer supplying supplement has accepted limited liability related to the manufacturing and original packaging of the study drug and to the losses, damages, claims or liabilities incurred by study participants based on known or unknown Adverse Events which arise out of the manufacturing and original packaging of the study drug, but not where there is any modification to the study drug (including without limitation re-packaging and blinding).

15 REPORTING, PUBLICATIONS AND NOTIFICATION OF RESULTS

15.1 AUTHORSHIP POLICY

Ownership of the data arising from this study resides with the study team. On completion of the study, the study data will be analysed and tabulated, and a clinical study report will be prepared in accordance with ICH guidelines.

15.2 PUBLICATION

The clinical study report will be used for publication and presentation at scientific meetings. Investigators have the right to publish orally or in writing the results of the study.

Summaries of results will also be made available to Investigators for dissemination within their clinics (where appropriate and according to their discretion).

15.3 PEER REVIEW

The study has been internally peer reviewed.

References

1. Giudice LC, Kao LC. Endometriosis. Lancet. 2004 364(9447):1789-99.

2. Farquhar C. Endometriosis. BMJ. 2007 334(7587):249-53.

3. Bulun SE. Endometriosis. N Engl J Med. 2009 360(3):268-79.

4. Gazvani MR, Smith L, Haggarty P, Fowler PA, Templeton A 2008. High omega-3: omega-6 fatty acid ratios and stromal cell cultures from women with and without endometriosis. Fertil Steril. 2001 76(4):717-22

5. Netsu S, Konno R, Odagiri K, Soma M, Fujiwara H, Suzuki M. Oral eicosapentaenoic acid supplementation as possible therapy for endometriosis. Fertil Steril. 2008 90(4 Suppl):1496-502).
